# Panos-Fermented Extract-Mediated Nanoemulsion: Preparation, Characterization, and In Vitro Anti-Inflammatory Effects on RAW 264.7 Cells

**DOI:** 10.3390/molecules27010218

**Published:** 2021-12-30

**Authors:** Rui Zhang, Esrat Jahan Rupa, Siwen Zheng, Jinnatun Nahar, Deok Chun Yang, Se Chan Kang, Yingping Wang

**Affiliations:** 1 State Local Joint Engineering Research Center of Ginseng Breeding and Application, Jilin Agricultural University, Changchun 130118, China; zr99101@163.com (R.Z.); siwen0717@126.com (S.Z.); 2 Department of Oriental Medicinal Biotechnology, College of Life Science, Kyung Hee University, Yongin-si 17104, Korea; eshratrupa91@gmail.com (E.J.R.); jinnatunnaharbph@gmail.com (J.N.); dcyang@khu.ac.kr (D.C.Y.)

**Keywords:** panos, nanoemulsion, anti-inflammatory, ultrasonication, ROS generation, NO production

## Abstract

This study focused on developing Panos nanoemulsion (P-NE) and enhancing the anti-inflammatory efficacy for the treatment of inflammation. The effects of P-NE were evaluated in terms of Nitric oxide (NO production) in Lipopolysaccharide (LPS), induced RAW 264.7 cells, Reactive oxygen species (ROS) generation using Human Keratinocyte cells (HaCaT), and quantitative polymerase chain reaction (qPCR) analysis. Sea buckthorn oil, Tween 80, and span 80 were used and optimize the process. Panos extract (P-Ext) was prepared using the fermentation process. Further high-energy ultra-sonication was used for the preparation of P-NE. The developed nanoemulsion (NE) was characterized using different analytical methods. Field emission transmission electron microscopy (FE-TEM) analyzed the spherical shape and morphology. In addition, stability was analyzed by Dynamic light scattering (DLS) analysis, where particle size was analyzed 83 nm, and Zeta potential −28.20 ± 2 (mV). Furthermore, 90 days of stability was tested using different temperatures conditions where excellent stability was observed. P-NE are non-toxic in (HaCaT), and RAW264.7 cells up to 100 µg/mL further showed effects on ROS and NO production of the cells at 50 µg/mL. The qPCR analysis demonstrated the suppression of pro-inflammatory mediators for (Cox 2, IL-6, IL-1β, and TNF-α, NF-κB, Ikkα, and iNOS) gene expression. The prepared NE exhibited anti-inflammatory effects, demonstrating its potential as a safe and non-toxic nanomedicine.

## 1. Introduction

The term “Panos” comes from the Greek word “Pan” meaning everything and “Axos” meaning heal/cure. Panos is a fermented extract prepared from the combined extract of *Panax ginseng*, *Dendropanax morbifera*, *Acanthopanax senticosus*, and *Kalopanax septemlobus*. Among them, *Panax ginseng* is considered the king of herbal medicine. Panax ginseng contains the most valuable ginsenoside and polyphenol that already evidenced for anti-cancer, anti-oxidant, anti-diabetic, anti-inflammatory, cardiovascular diseases, and stimulant of immune system activities [1,2,3,4]. In addition, the *Dendropanax morbifera* contains the most-reported chemicals: acetylene, saponin glycosides, oleifoliosides A, dendropanoxide, terpenoid, and oleifoliosides [5,6], also traditionally used for the treatment of high blood pressure, anti-coagulant, anti-cancer, and anti-inflammatory diseases [7,8,9]. *Acanthopanax senticosus* and *Kalopanax septemlobus* also contain valued medicinal components. The highly valued medicinal components contain lipophilic constituents that the body cannot absorb and are entirely excreted with urine. The NE has lipophilic components’ encapsulation ability that enhances the therapeutic drug efficacy and well absorbs into the body [10].

Recently, NE has been widely used to protect the active components of plants from extreme conditions, enhance drug solubility, stability, and increase drug efficacy [11]. The encapsulation of active constituents is a novel delivery approach for NE that can entrap a higher quantity of drugs compared to conventional preparation [12], and are classified as O/W type (oil is dispersed in aqueous phase), bicontinuous (water and oil are interspersed into the system), and W/O (water is dispersed in oil phase). The approach of NE offers various advantages over other dosages forms, namely, delivery of lipophilic drugs [13]; increase the rate of absorption [14]; reduce oxidation and hydrolysis in O/W NE [15]; helping to soluble water-insoluble drugs, reducing doses and side effects [16]; and working as a non-irritant vehicle for skin mucous membrane delivery [17].

NE is widely used for the treatment of various diseases like inflammation. Inflammation is an auto reaction of the body against diseases curing organisms expressed as a cellular injury that induces the immune system mediators. However, the excessive inflammatory response is responsible for chronic inflammation damaging the normal tissue. Several mediators IL-1α, IL-1β, IL-6, and tumor necrosis factor-alpha (TNF-α) [18], are the key mediator of skin inflammation responses. Therefore, treatment can reduce the overproduction of pro-inflammatory mediators, effectively treating inflammation. The commercial drugs in the market are mainly reported as having low solubility and poor absorption [19] in the body, so alternative vehicles like NE can increase the drug efficacy and reduce the side effect. In addition, ginsenoside is also reported as having low solubility in the body [2]. Therefore, the natural product-based drug can be the best choice for overcoming all problems. This article focuses on developing oil in water NE (O/W) using Panos extract, plant oil, and surfactant (non-ionic, which is widely used in industries). An ultrasound method was recommended due to better encapsulation, higher yield, less energy, control delivery, and better droplet size than any other conventional way [20]. NE possesses more highly kinetic stability, due to the small droplet size ranges 50–200 nm, than other conventional emulsions [21].

The valued medicinal constituents from the Panax genus are mostly insoluble in water; therefore, Panos extract was fermented and developed oil in water (W/O) nanoemulsion using the ultra-sonication method for entrapment of hydrophobic (water-insoluble) components of Panos with oil to enhance medicinal value. This research believes that P-NE can be a potential nano-drug for inflammation diseases.

## 2. Results and Discussion

### 2.1. P-NE Preparation

The P-NE was prepared with some minor modifications [22]. The sea buckthorn fruit oil was used as an oil phase due to its high medicinal values, and it also has been reported as a folk medicine in China and Korea for its anti-oxidant, anti-inflammation, and antibacterial properties [23,24,25]. Generally, NE can be classified according to the percentage of water, oil, and surfactant; the surfactant (1.5–10%) and the water (50~92%). The surfactant percentage is considered in specific ranges because too much can form precipitation and skin irritation [26]. This study followed the surfactant concentration of 6%, where a mixed surfactant was used to encapsulate the hydrophobic constituents on the oil phase. Span 80 containing the HLB value 4.3 is considered a highly lipophilic surfactant, and tween 80 with an HLB value of 15 is regarded as highly hydrophilic. Therefore, during NE preparation, a mixed surfactant was recommended. The HLB values were calculated by mixing different surfactant ratios and optimizing the mixture to find a stable ratio (Table 1). Optimization results exposed that 7:3 is highly stable under visual observation, which contains an HLB value of 11.79. The prepared sample did not show any phase separation, creaming, or precipitation during the observation period that highly recognized its stability [27]. The optimized samples (S1, S2, S3, and S4) are displayed in Table 2. Overall, the optimization results indicated the S2 sample is stable up to (7 days) where other samples showed some creaming and S2-contains 87% water and 8% oil, and surfactant 5%. Finally, S2 is considered for further experiments due to its higher stability.

### 2.2. Long-Term Stability Test

**Figure 1:**
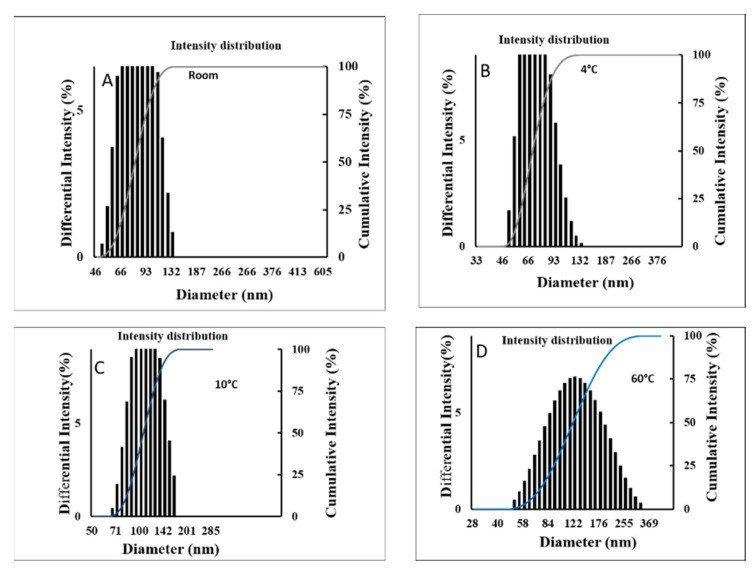
DLS analysis analyzed the size of Panos nanoemulsion indifferent stored temperatures (**A**) Room temperature (**B**), 4 °C (**C**), 10 °C (**D**).

The stable NE were sent to evaluate long-term stability in different temperatures, and storage times are shown in (Table 3). Sample S2 was screened for initial size, PDI value, zeta potential, and different pH levels. All parameters were checked after three months of storage at (4 °C, 10 °C, and 60 °C). After 90 days of storage time, results indicated that at 60 °C temperature storage, little NE creaming formed. In-room temperatures of 4 °C and 10 °C storage did not show any phase separation or creaming properties. At high temperatures, NE showed creaming due to the use of non-ionic surfactant. It was reported that the non-ionic surfactant is heat-sensitive; therefore, it can form creaming [28]. The DLS analysis analyzed the size, Zeta potential, and PDI value of the NE. In the (4 °C, 10 °C, and 60 °C) storage conditions, the size calculated 88 ± 2, 117 ± 2, 147 ± 1 nm, respectively shown in Figure 1. The PDI value also did not change much, being about 0.100 ± 0.010, 0.102 ± 0.02, and 0.115 ± 0.02, respectively. The stability of NE can understand by analyzing the zeta potential value. The different storage conditions (4 °C, 10 °C, and 60 °C), the zeta potential value is about −27.14 ± 0.7, −21.81 ± 0.5, and −14 ± 1.2 mV, respectively (Table 3). Mostly for NE stability, more than ±20 mV is considered stable, and 4–5 pH is considered safe for skin absorption [10]. The zeta-potential value is about −27 mV, and the PDI value is 0.100 at 4 °C storage conditions. The polydispersity index <0.250 is considered monodispersed. Here, P-NE showed a 0.100 PDI value, which is considered a uniform formulation [29]. In addition, the non-ionic surfactant can form a negative zeta potential that is enough to make a stable barrier into the droplet.

### 2.3. FT-IR Analysis

**Figure 2:**
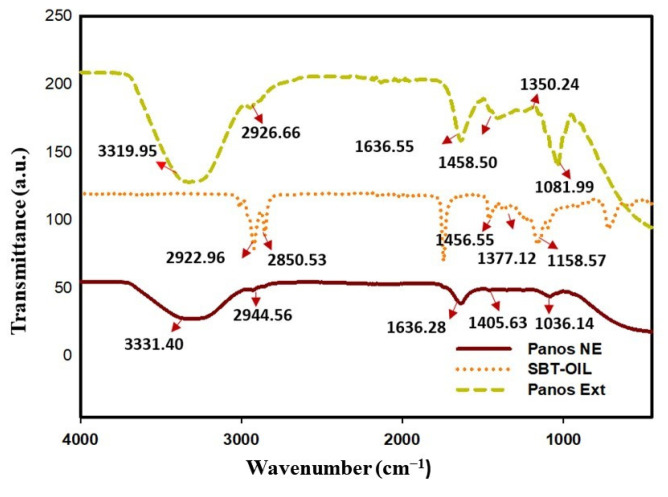
FT-IR spectra of Panos nanoemulsion show Panos NE (Red line), Panos extract (Green line), and Sea buckthorn oil (Yellow line).

The FT-IR spectra of P-Ext and NE are shown in (Figure 2). The absorption band in P-NE is represented at 3319, 2926, 1636, 1458, 1350, and 1080 cm^−1^. The responsible functional group in those bands are phenolic (-OH) and Alkyne (C=O) stretch (amide bond), respectively. The major absorption band for P-Ext is shown in 3331, 2944, 1636, and 1405 cm^−1^, respectively. This major band is responsible for the major functional group of Phenolic (-OH), C=O stretch (amide), Amides I (N-H bending), ester carbonyl (C-O-C stretch), and polysaccharides, respectively. The sea buckthorn oil also showed the absorption band in a specific region at 2922, 2850, 1456, and 1377 cm^−1^. This absorption band, responsible for the functional group, is antisymmetric CH3 and CH2 scissoring and antisymmetric CH_2_, respectively.

### 2.4. FE-TEM Analysis

**Figure 3:**
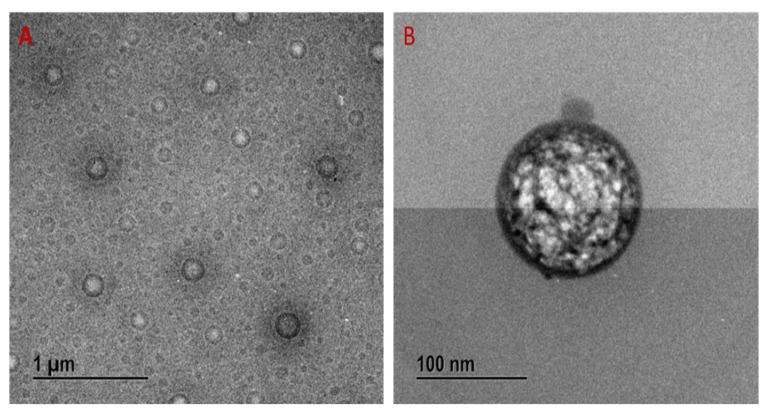
FE-TEM images of Panos nanoemulsion, (**A**) 1 µm scale bar; (**B**) 100 nm.

The shape and morphology of the P-NE were identified using FE-TEM analysis. The NE is spherical, and the size is well-matched with DLS analysis. The TEM images demonstrated that the spherical shape NE is well distributed, shown in Figure 3A (1 µm scale bar) and 3B (100 nm Scale bar).

### 2.5. Cell Viability Analysis

**Figure 4:**
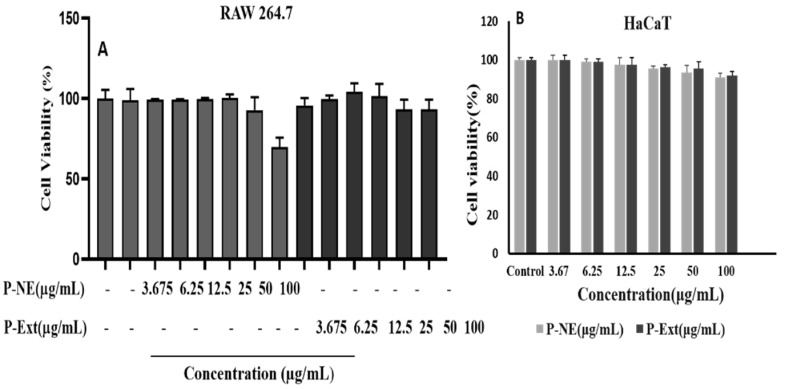
Effect of Panos nanoemulsion on cell viability in (**A**) RAW 264.7; (**B**) HaCaT. Cell viability was assessed in different concentrations of Panos nanoemulsion in 24 h using the MTT assay.

The cell cytotoxicity was analyzed using the Murine macrophage (Raw 264.7) and Human keratinocytes cells (HaCaT). The cell cytotoxicity analysis in the normal cell can give information about the drug’s safety on human application and indicate the drug’s doses. The normal cell line’s toxicity effect was measured using MTT assay in different concentrations of P-NE (control, 3.67, 6.25, 12.5, 25, 50, and 100) µg/mL for 24 h. Results indicated that P-NE is non-toxic for human cells until 100 μg/mL, where 96% of the cell is viable in the HaCaT cell line in Figure 4B. In addition, NE was checked for Raw 264.7 cells where 100 μg/mL concentrations are non-toxic shown in Figure 4A. Toxicity effect was measured using MTT assay in different concentrations of P-NE (control, 3.67, 6.25, 12.5, 25, 50, and 100) µg/mL for 24 h. Results indicated that P-NE is non-toxic for human cells until 100 μg/mL, where 96% of the cell is viable in the HaCaT cell line in Figure 4B. In addition, NE was checked for Raw 264.7 cells where 100 μg/mL concentrations are non-toxic shown in Figure 4A.

### 2.6. Effect of P-NE on NO Production and ROS Generation

**Figure 5:**
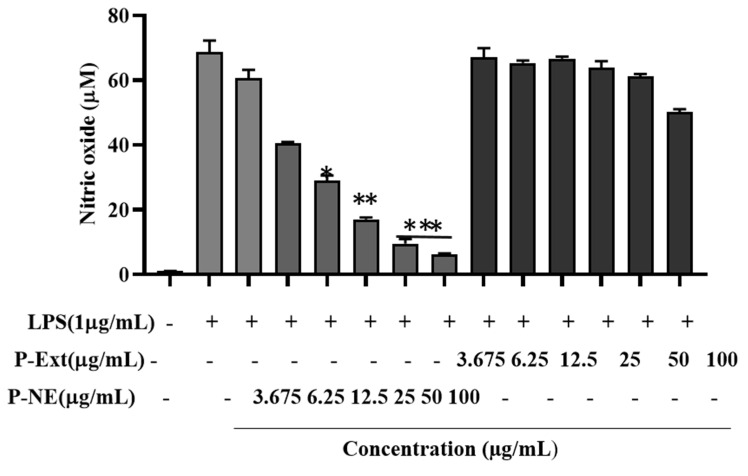
The effects of Panos nanoemulsion on NO production were assessed by 1 µg/mL LPS induced RAW 264.7 cells. Data presented as ±SEM, * *p* < 0.05 ** *p* < 0.01 vs. normal control. All treatment was performed three times.

**Figure 6:**
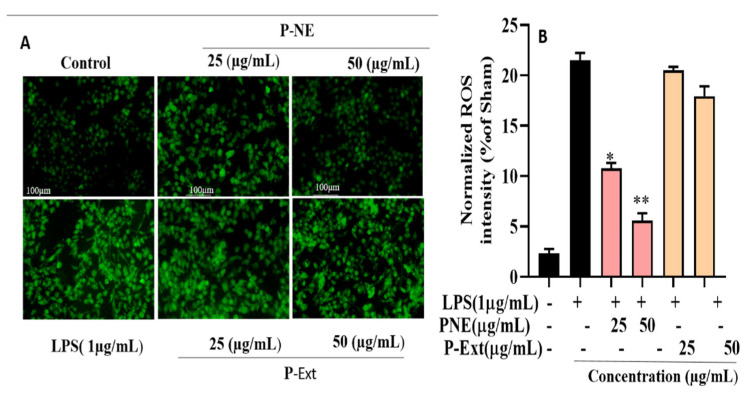
Cells were stained with (**A**) Superoxide detection assay kit/ROS using 200 magnification at fluorescence microscope and (**B**) Statistics analysis of fluorescence intensity of ROS staining. Data presented as ±SEM, * *p* < 0.05 ** *p* < 0.01 vs. LPS treated group. All treatment was performed three time.

The macrophage is considered the primary initiator for pathological inflammation, chronic inflammation, and autoimmune diseases [28]. P-NE and extract were both treated in two different concentrations (25, 50) µg/mL as a pre-treatment. Nanoemulsion significantly inhibited the LPS-induced nitrite oxide level in macrophages without toxicity Figure 5. This study results highlight that P-NE inhibited the NO production; it may be due to the well binding of lipophilic–hydrophilic components into the NE droplet. Previous research suggests that medicinal ingredients’ binding ability of NE from extracts increases due to the use of non-ionic surfactant and also for better absorption of the drug, the size is a crucial factor [28]. The P-Ext contains different ginsenosides that have already been reported the ability to reduce NO [29]. Moreover, the sea buckthorn oil contains alpha-tocopherol, which has already been reported to reduce inflammation diseases [30]. Overall, the synergistic effect of P-Ext and sea buckthorn oil helps to reduce NO release of macrophages initiation of inflammation and progression of autoimmune diseases. Macrophages are also responsible for auto-inflammatory illnesses like cardiovascular and rheumatoid arthritis [28]. ROS generation and oxidative stress are crucial factors for inflammatory diseases. According to previous reports, NE using pomegranate seed oil seeds has shown excellent anti-inflammation ability [31]. Moreover, excessive production of ROS can contribute to the development of cancer [32]. In this, study results indicated that ROS secretion enhanced 77% after LPS treatment. The P-NE at pre-treatment with (25, 50) μg/mL significantly reduced the ROS around 40 and 64%, respectively shown in Figure 6. The previous study also suggested similar results that P-Ext contains medicinal components that have anti-inflammatory effects [33]. P-NE significantly reduces the ROS generation compared to P-Ext; due to the size and entrapment of active components into the droplet, it increases the drug absorption into the cells. These preliminary results suggested that P-NE has anti-inflammatory effects, directly suppressing the LPS induced cell or inhibiting the NF-κB signaling pathway.

### 2.7. qPCR Analysis on Pro-Inflammatory Mediators

**Figure 7:**
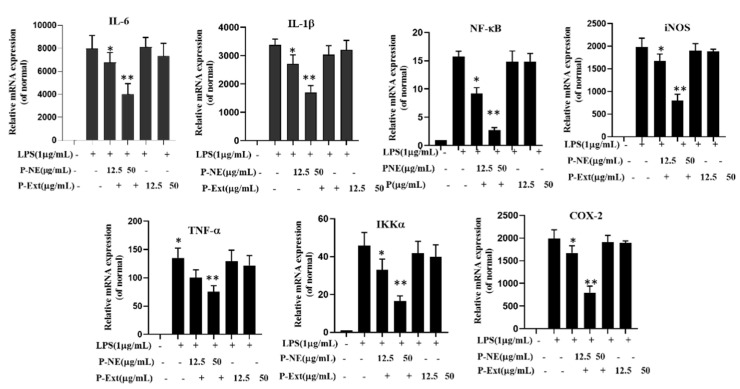
Effects of Panos nanoemulsion on pro-inflammatory mediators IL-6, IL-1β, NF-κB, iNOS, TNF-α, IKKα, and Cox-2 in LPS induced RAW 264.7 cells. The mRNA expression was determined by q-PCR analysis. Data presented as ±SEM, * *p* < 0.05 ** *p* < 0.01 vs. normal. All treatment was performed three times.

The pro-inflammatory mediators have a crucial effect on inflammatory diseases. The overexpression of these factors, such as nitric oxide synthase (iNOS), tumor necrosis factor-a (TNF-α), cyclooxygenase-2 (Cox-2), interleukin (IL-1β), and IL-6, by macrophages, is responsible for various chronic inflammatory diseases. Therefore, this study focused on investigating the P-NE-affected gene expression on those factors. The Raw 264.7 cells were pretreated with P-NE and P-Ext at (25, 50) μg/mL for 1 h. Then, induced by LPS for 24 h is shown that LPS increases the mRNA expression of IL-6, IL-1β, NF-κB, iNOS, TNF-α, IKKα, and Cox-2, expression by 78.0, 64.3, 64.3, 57.7, 59.4, 62.4, and 65.4% respectively, compared untreated cells (Figure 7). The P-NE treated cell significantly suppressed the gene expression of 39.0, 32.2, 35.7, 55.9, 36.6, 33.9, and 28.7%, respectively shown in Figure 7. P-Ext contains medicinal compounds like ginsenoside, rutin, and polyphenol that have already been proven for strong anti-inflammatory efficacy [34]. Therefore, the effective inhibition of NO and mRNA may occur due to the size and entrapment of active components into the droplet and increases the drug absorption into the cells.

## 3. Materials and Methods

P-Ext was prepared using the fermentation method where *Panax ginseng*, *Dendropanax morbifera*, *Acanthopanax senticosus*, and *Kalopanax septemlobus* were used collected from the lab of Hanbang bio, Suwon, Korea. Sea buckthorn oil (vitamin fruits oil) was collected from China. NaOH and HCl, Lactic acid, were purchased from Dae-Jung chemical co Pyeontaek, Korea. Ultrasonic probe (3000, India, probe diameter 3 mm), Tween 80 and span 80 both purchased from sigma Aldrich U.S.A. Fetal bovine serum (FBS) and penicillin-streptomycin solution were purchased from Gen DEPOT (Barker, TX, USA). Dulbecco’s Modified Eagle’s Medium (DMEM), high glucose, pyruvate was purchased from Gibco (Waltham, MA, USA). (3-(4, 5-dimethylthiazol-2-yl)-2,5-diphenyltetrazolium bromide or MTT) the solution brought from Life Technologies, suwon, Korea.

### 3.1. P-Ext Preparation

The fermented extract was prepared using different ratios of *Panax ginseng, Dendropanax morbifera, Acanthopanax senticosus,* and *Kalopanax septemlobus*. The dried four samples were extracted hot water extraction process at 90 °C for 8 h. The filtered extracts were concentrated under reduced pressure (800–850 mm/Hg) at 60 °C to form 60 Brix. The concentrated extract was diluted with water into 10 Brix. The calculated ratio was PG (10 Brix: DP (10 Brix): AP (10 Brix): KP (10 Brix): 4:1.5:1.5:3 (Final weight was 1 kg). The final weight was fermented with enzymes (viscozyme 50 mL + Pectinex 50 mL) at 55 °C for 24 h. Further, the final concentration was reduced to 30 Brix using reduced pressure at 60 °C temperature for future experiments.

### 3.2. NE Preparation

P-NE was prepared using P-Ext, sea buckthorn oil, and different surfactants ratios (Tween 80 and Span 80). A total four types of samples were prepared and optimized. Moreover, the HLB value was calculated for two surfactant mixtures to analyze the mixture’s stability. HLB value calculated using the Griffin method

(1) $$\mathrm{HLB}\;\mathrm{value}\;=\frac{\mathrm{HLB}\;\mathrm{value}\;1\times \mathrm{surfactant}\;1\left(\%\right)+\mathrm{HLB}\;\mathrm{value}\;2\times \mathrm{surfactant}\;2\left(\%\right)}{\mathrm{Surfactant}1\left(\%\right)+\mathrm{surfactant}\;2\left(\%\right)}$$

HLB value 1 = surfactant 1 HLB value, HLB value 2 = surfactant 2 HLB value, Surfactant 1 (%) = how much surfactant was taken, Surfactant 2 (%) = how much surfactant taken

This study used Tween 80 and Span 80 surfactant mixture ratio.

Total four samples (S1, S2, S3, and S4) was prepared using different concentration of P-Ext, surfactant, and oil. All sample was optimized and checked their stability during processing time. The coarse emulsion was prepared, following 87 mL water with 10 g P-Ext at room temperature. Sea buckthorn oil and surfactant mixture was added with constant stirring 500 rpm (Tween 80:span 80), maintaining 7:3 proportion (HLB value calculated at 11.79). The prepared coarse emulsion was transferred to the ultrasonic probe to create the droplet cavitation. This study maintained the sonic frequency at 20 kHz with 130 w, amplitude (50%) with a 10 s pulse. The time was optimized, and the probe used for creating cavitation was 3 mm (titanium alloy). The whole ultra-sonication time sample was covered with an ice bath to avoid thermal damage to the NE. Four sets of NE were prepared using the optimized conditions and checked their stability with visual observation at seven days. Further, the best-optimized sample was chosen for a future experiment.

### 3.3. Characterization of NE

The characterization process was carried out using different analytical methods for analyzing morphology, size, and stability. The Field Emission Transmission Electron Microscope (FE-TEM) analyzer (JEM-2100 F, JEOL, Peabody, MA, USA) was used to evaluate the NE’s morphology and shape. One drop of the sample was deposited into the copper grid and dried in a 60-degree oven for ten minutes during the analysis of the sample. The dynamic light scattering (DLS; Otsuka Electronics, Shiga, Osaka, Japan) analyzer analyzed the sample’s particle size and zeta potential. The prepared NE was diluted ten times with distilled water to explore the particle size, zeta potential, and PDI (polydispersity index) value of the materials. The maintained temperature was 25 °C, and as a reference condition, a dispersive medium of pure water with a refractive index of 1.3328, a dielectric constant of 78.3 viscosity of 0.8878 was used. The Fourier-transform infrared spectroscopy (FT-IR) analysis of the Perkin Elmer Spectrum FT-IR spectrometer (Boston, MA, USA) was used to identify the functional group of NE that bonded from the extract in the range of 4000–450 cm^−1^.

### 3.4. Stability Determination

The stability of NE was checked at a period of time (90 days) stored in different temperatures (Room, 4 °C, 25 °C, and 60 °C) to analyze the zeta potential, particles size, and PDI value. In addition, the visual observation (creaming, Phase separation) was monitored in the optimization time.

### 3.5. Measurement of Nitric Oxide (NO) Production in LPS Induced Raw 264.7 Cell Line

The RAW264.7 cell was seeded at seeding concentration 1 × 10^4^ cells/well. At first, the sample was pretreated with different concentrations [20]. After 1 h, the cell was stimulated with LPS (1 μg/mL) collected from (Sigma-Aldrich, St. Louis, MO, USA). The cell supernatant was collected after 24 h of incubation. NO (Nitric oxide) production was measured using Griess reagent by colorimetric Griess reaction (100 mL of pre-mixed Griess reagent from Sigma-Aldrich, St. Louis, MO, USA). The equal volume of reagent was added with supernatant and incubated for 10 min to determine NO production. Two multimode microplate readers were used to measure the absorbance at 450 nm for Nitric oxide production assay.

### 3.6. Quantitative PCR Analysis

The RAW264.7 cells were pretreated with NE, and after the one-hour cell was treated with LPS (1 μg/mL) incubated for 24 h. The Total RNA was extracted from cells using Trizol reagent kit instructions (Invitrogen, Carlsbad, CA, USA). At first, 2000 ng of RNA was reversed transcribed with oligo dt _(15)_ primer (AccuPower RT PreMix, Bioneer Co., Daejeon, Korea) and reversed transcriptase. The PCR pre-mix from AccuPower HotStart (Bioneer Co., Korea) was used for PCR amplification. The 2.0% agarose gel electrophoresis and staining with ethidium bromide dye were performed for the separation of PCR products, finally detected by UV-illuminator. The housekeeping gene glyceraldehyde-3-phosphate dehydrogenase (GAPDH) was used. In (Table 4) the primer sequence is presented.

### 3.7. Cell Viability Analysis

In vitro cell viability assay was performed using the previous method with minor modification [22]. A total of 10% FBS (Fetal bovine serum) 1% penicillin/Streptomycin (p/s) (WElGENE Inc., Daegu, Korea) was used for the culture of the cell. The Dulbecco’s modified eagle’s medium (DMEM), collected from Gibco-Grand Island, NY, USA, was used to culture the cell. The HaCaT (immortal keratinocyte cell line) and raw 264.7 cells (murine macrophage cell line) were collected from (KCLB, Seoul, Korea). Cell-maintained conditions were incubated at 37 °C in a humidified atmosphere containing 5% CO_2_ and 95% air. The cell was seeded in a 96 well plate at 1 × 10^4^ seeding concentration. The different concentration (C, 10, 25, 50, 75, and 100) µg/mL of Panos sample and NE was treated to both the cell line for 24 h incubation. About 20 µL of MTT (3-(4, 5-dimethyl-2-thiazolyl)-2, 5-diphenyl-2H tetrazolium bromide from Life Technologies, Eugene, OR, USA, was used for performing MTT analysis. After adding the MTT, the solution cell was incubated at 37 °C for 4 h. Finally, 100 µL DMSO was added to each well, and a reading was taken in 570 nm with an Enzyme-Linked Immunosorbent Assay (ELISA) reader (Bio-Tek, Instruments, Inc., Winooski, VT, USA). The viable cell makes the MTT solution violet color.

### 3.8. Reactive Oxygen Species (ROS) Assay

Panos NE at (25, 50) µg/mL concentration was treated to the LPS (1 μg/mL) induced Raw 264.7 cells to release the intercellular ROS. The Cellular ROS/Superoxide detection assay Kit (Cambridge, MA, USA, Ex/Em (490/525 nm) was used to detect ROS generation in the induced cell. The fluorescence image was measured using the LSM 510 and 510 META laser scanning microscope.

## 4. Conclusions

This study highlighted that the Panos nanoemulsion serves as a potential anti-inflammatory agent in RAW 264.7 cell lines. It was found that Panos nanoemulsion inhibits NO production and intercellular ROS generation and suppresses the pro-inflammatory mediators. Moreover, the small sizes and spherical shapes of the prepared nanoemulsion exhibited good penetration into the cell; therefore, nanoemulsion shows a significant anti-inflammatory effect, better than the Panos extract. The excellent stability of Panos nanoemulsion declares it as a potential candidate for the treatment of inflammation; however, further investigations are needed at the in vivo level.

## Figures and Tables

**Table 1:** Optimization of HLB value for tween 80 and span 80 mixture.

Surfactant (Mixed)	Composition	HLB Value
Tween 80: Span 80	9:1	13:93
Tween 80: Span 80	8:2	12:86
Tween 80: Span 80	7:3	11:79
Tween 80: Span 80	6:3	10:2

**Table 2:** The optimization chart of P-NE.

Scheme 2.	P-Ext (% H_2_O)	Surfactant	Oil
S1	5 g (86% H_2_O)	6%	8%
S2	10 g (87% H_2_O)	5%	8%
S3	15 g (92% H_2_O)	3%	5%
S4	30 g (87% H_2_O)	7%	6%

**Table 3:** Stability analysis of P-NE during 90 days storage.

No. of Parameter	24 h		90 Days (After)	
Temperature (°C)	Room temp	4 °C	10 °C	60 °C
Size (nm)	83.0 ± 2.1	88.1 ± 3	117.5 ± 2	147.8
PDI value	0.078 ± 0.023	0.100 ± 0.010	0.102 ± 0.02	0.115 ± 0.02
Zeta potential (mV)	−28.20 ± 2	−27.14 ± 0.7	−21.81 ± 0.5	−14 ± 1.2
pH value	6.43 ± 0.22	6.34 ± 0.23	6.49 ± 0.07	5.23 ± 0.06

**Table 4:** Primer sequence for the inflammatory gene.

Primer	Primer Sequence	
GAPDH	Forward	5′-ACCACAGTCCATGCCATCAC-3
	Reverse	5′-CCACCACCCTGTTGCTGTAG-3
IL-6	Forward	5′-AGCCCACGTCGTAGCAAACCACCAA-3′
	Reverse	5′-AACACCCATTCCCTTCACAGAGCAAT-3′
IL-1β	Forward	5′-TGCAGAGTTCCCCAACTGGTACATC-3′
	Reverse	5′-GTGCTGCCTAATGTCCCCTTGAATC-3′
NF-κB	Forward	5′-TATTTCAACCACAGATGGCACTGC-3
	Reverse	5′-CAGATTTTGACCTGAGGGTAAGAC-3
iNOS	Forward	5′-AATGGCAACATCAGGTCGGCCATCACT-3
	Reverse	5′-GCTGTGTGTCACGAAGTCTCGAACTC-3
TNF-α	Forward	5′-AGCCCACGTCGTAGCAAACCACCAA-3′
	Reverse	5′-AACACCCATTCCCTTCACAGAGCAAT-3′
IKKα	Forward	5′-GGCCTGTGATGTCCTGAAGAATT-3
	Reverse	5′-TCGAATCCCAGACCCTATATCACT-3

## Data Availability

Not applicable.

## Sample Availability

We wish not to publicly share the available data, as further research is going on with this basic research.
